# Status and methodology of publicly available national HIV care continua and 90-90-90 targets: A systematic review

**DOI:** 10.1371/journal.pmed.1002253

**Published:** 2017-04-04

**Authors:** Reuben Granich, Somya Gupta, Irene Hall, John Aberle-Grasse, Shannon Hader, Jonathan Mermin

**Affiliations:** 1 International Association of Providers of AIDS Care, Washington, D.C., United States of America; 2 Centers for Disease Control and Prevention, Atlanta, Georgia, United States of America; University of Berne, SWITZERLAND

## Abstract

**Background:**

In 2014, the Joint United Nations Program on HIV/AIDS (UNAIDS) issued treatment goals for human immunodeficiency virus (HIV). The 90-90-90 target specifies that by 2020, 90% of individuals living with HIV will know their HIV status, 90% of people with diagnosed HIV infection will receive antiretroviral treatment (ART), and 90% of those taking ART will be virally suppressed. Consistent methods and routine reporting in the public domain will be necessary for tracking progress towards the 90-90-90 target.

**Methods and findings:**

For the period 2010–2016, we searched PubMed, UNAIDS country progress reports, World Health Organization (WHO), UNAIDS reports, national surveillance and program reports, United States President’s Emergency Plan for AIDS Relief (PEPFAR) Country Operational Plans, and conference presentations and/or abstracts for the latest available national HIV care continuum in the public domain. Continua of care included the number and proportion of people living with HIV (PLHIV) who are diagnosed, on ART, and virally suppressed out of the estimated number of PLHIV. We ranked the described methods for indicators to derive high-, medium-, and low-quality continuum. For 2010–2016, we identified 53 national care continua with viral suppression estimates representing 19.7 million (54%) of the 2015 global estimate of PLHIV. Of the 53, 6 (with 2% of global burden) were high quality, using standard surveillance methods to derive an overall denominator and program data from national cohorts for estimating steps in the continuum. Only nine countries in sub-Saharan Africa had care continua with viral suppression estimates. Of the 53 countries, the average proportion of the aggregate of PLHIV from all countries on ART was 48%, and the proportion of PLHIV who were virally suppressed was 40%. Seven countries (Sweden, Cambodia, United Kingdom, Switzerland, Denmark, Rwanda, and Namibia) were within 12% and 10% of achieving the 90-90-90 target for “on ART” and for “viral suppression,” respectively. The limitations to consider when interpreting the results include significant variation in methods used to determine national continua and the possibility that complete continua were not available through our comprehensive search of the public domain.

**Conclusions:**

Relatively few complete national continua of care are available in the public domain, and there is considerable variation in the methods for determining progress towards the 90-90-90 target. Despite bearing the highest HIV burden, national care continua from sub-Saharan Africa were less likely to be in the public domain. A standardized monitoring and evaluation approach could improve the use of scarce resources to achieve 90-90-90 through improved transparency, accountability, and efficiency.

## Abstract 2

In a systematic review, Reuben Granich and colleagues assess the quality and comparability of publicly available data on national HIV care continua and progress towards the 90-90-90 targets.

## Author summary

**Why was this study done?:**

- Treatment prevents human immunodeficiency virus (HIV) illness, death, and transmission, prompting the Joint UN Program on HIV/AIDS (UNAIDS) to issue the 90-90-90 target.
- The 90-90-90 target specifies that by 2020, 90% of individuals living with HIV will know their HIV status, 90% of people with diagnosed HIV infection will receive antiretroviral treatment (ART), and 90% of those taking ART will be virally suppressed.
- Our review aims to answer three critical questions: (1) What data for national continua of care are available in the public domain? (2) What is the quality and comparability of the information presented? and (3) How close are we to achieving the UNAIDS 90-90-90 targets?

**What did the researchers do and find?:**

- For the period 2010–2016, we searched the public domain for the latest available national HIV care continuum with the number and proportion of people living with HIV (PLHIV) who are diagnosed, on ART, and virally suppressed.
- We found 53 national care continua with viral suppression estimates (representing 54% of the 2015 global estimate of PLHIV), and the average proportion of PLHIV on ART and the average proportion of PLHIV who were virally suppressed were 48% and 40%, respectively.
- Although seven countries (Sweden, Cambodia, UK, Switzerland, Denmark, Rwanda, and Namibia) had reached or were within 12% and 10% of achieving the 90-90-90 target for “on ART” and for “viral suppression,” only 9 countries in sub-Saharan Africa had care continua with viral suppression estimates in the public domain.
- Limitations include significant variation in the methods used to determine national continua and the possibility that complete continua were not available through our comprehensive search of the public domain.

**What do these findings mean?:**

- Many countries are progressing toward and will likely achieve 90-90-90; however, it will be important to use consistent and accurate methods to report on progress.
- Relatively few complete national continua of care are available in the public domain, and there is wide variation in the methods for determining progress towards the 90-90-90 target.
- Despite bearing the highest HIV burden, complete national care continua from sub-Saharan Africa were less likely to be in the public domain.
- A standardized monitoring and evaluation approach could improve the use of scarce resources to achieve 90-90-90 through improved transparency, accountability, and efficiency.

## Introduction

In 2003, the World Health Organization (WHO) “3 by 5” initiative called for providing 3 million people living with HIV in low- and middle-income countries with antiretroviral treatment (ART) by the end of 2005 [1]. Despite considerable skepticism regarding the advisability and feasibility of expanding access to ART, by June 2016 around 18.2 million, or 49% of people living with HIV, were taking ART [2]. Efforts to expand access to treatment are supported by scientific evidence that treatment prevents illness and death, reduces human immunodeficiency virus (HIV) transmission, and can decrease health care costs [3–6]. Ensuring access to care and successful viral suppression is a challenge. Although hundreds of millions of HIV tests have been performed, one of the most important barriers to earlier ART and viral suppression is limited HIV diagnosis among people living with HIV, of whom only 60% had been diagnosed as of 2015 [7]. Initiation of ART has primarily relied on CD4 cell count measurement, with access to the more useful viral load monitoring increasing only recently. The importance of accelerating access to diagnosis, treatment, and viral suppression as significant elements in ending the epidemic prompted the Joint UN Program on HIV/AIDS (UNAIDS) to release the HIV 90-90-90 target [8]. The target specifies that by 2020, 90% of individuals living with HIV will be diagnosed and know their HIV status, 90% of people with diagnosed HIV infection will receive sustained ART, and 90% of those on ART will be virally suppressed [8]. The target has been set as a minimum, with 95-95-95 by 2030 being envisioned as the target after 2020.

The establishment of the 90-90-90 target has placed increased emphasis on accurate monitoring and evaluation of the national continua of care from HIV diagnosis to viral suppression [9–15]. The importance of a standardized monitoring and evaluation framework was reflected in the 2001 UN General Assembly Special Session on HIV/AIDS that called for 13 core national-level indicators for monitoring progress [16]. In 2003, the US President’s Emergency Plan for AIDS Relief (PEPFAR) and the Global Fund Against AIDS, Tuberculosis and Malaria (GFATM) resulted in an increase in resources for the global HIV response [17–19]. Efforts to improve both accountability and impact prompted the allocation of hundreds of millions of dollars for monitoring and evaluation of the HIV response. However, despite the commitment by stakeholders to a standard country-level monitoring and evaluation system [16], there has been an unprecedented increase in the number, type, complexity, and costliness of data collection systems, in part due to the expansion of individual site-level systems for clinical and program management. Reflecting the complex HIV response, these data are diverse, gathered from a variety of stakeholders on a large number of interventions and outcomes for a variety of purposes. The multiplicity of monitoring and evaluation systems, inconsistent methodologies often within the same geographic area, and paucity of data in the public domain create significant challenges for understanding fundamental questions about the impact of the investment in addressing the HIV epidemic. Specifically, despite the significant investment of approximately 19 billion US dollars (USD) per annum in the global HIV response and successfully providing access to treatment for 18.2 million people by June 2016 [20], many countries still face significant challenges when determining their national HIV care continuum [9,13,14,17,21–23].

The 90-90-90 target focuses on improving the continuum of HIV care from diagnosis to viral suppression [8]. By specifying the proportion of diagnosis, ART, and viral suppression as important pillars of the HIV response, the target explicitly highlights the importance of providing treatment for nearly everyone and implicitly requires monitoring systems to measure progress. The indicators provide an important political and programmatic accountability framework and also emphasize the importance of achieving viral suppression through the continuum of care for at least 73% of people living with HIV by 2020. While there is growing consensus around the 90-90-90 target, measuring progress requires the ability to determine a country-level continuum of care that includes the proportions of all people living with HIV who are diagnosed with HIV, on ART, and virally suppressed [8,9]. Information regarding continua of care is limited, and efforts to describe global, national, and local continua of care have often used nonstandardized approaches or neglected to describe how the estimates were determined [23,24]. Pulling together the information is complicated by barriers that include the lack of a national cohort approach and individual patient identifiers, anonymous and unlinked testing and treatment services, and delays and loss to follow-up due to providing treatment only for those who are severely immunocompromised. As a result of these and other challenges, accurate national care continua from sub-Saharan Africa, the region with the highest HIV burden, are often unavailable or of uncertain quality. Our study aimed to answer these critical questions regarding data availability, quality, and progress toward 90-90-90: (1) What data for a complete officially endorsed national continuum of care are available in the public domain? (2) Of the national-level continuum data that are available, what are the quality and comparability of the information presented? How close are we to using a standard definition for measuring the continuum? and (3) How close are we to achieving the UNAIDS 90-90-90 targets? Which targets are proving to be the most challenging? How close are we to being able to measure the “third 90” (viral suppression)?

## Methods

**Fig 1:**
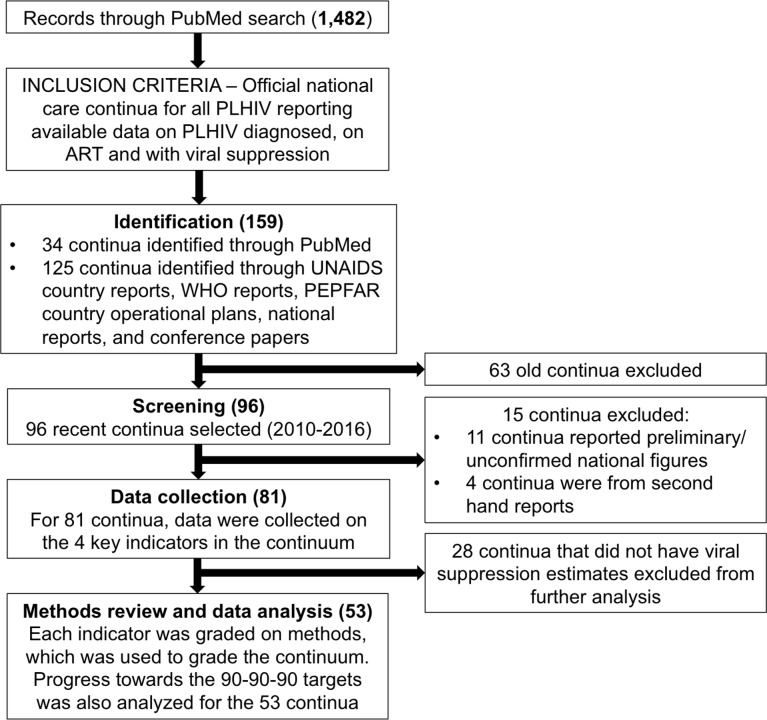
Search flow diagram. WHO, World Health Organization; UNAIDS, Joint UN Programme on HIV/AIDS; PEPFAR, the US President’s Emergency Plan for AIDS Relief. For 2010–2016, we searched PubMed, UNAIDS and WHO reports, national surveillance and program reports, PEPFAR 2016 Country Operational Plans, and conference presentations and/or abstracts for national HIV continua of care. The search strategy included the keywords (HIV OR AIDS) AND (treatment) AND (cascade OR continuum of care OR care continuum OR continua OR spectrum of care OR 90-90-90 OR viral suppression) for data published in the public domain. The search was designed to identify the most recent officially reported or sanctioned national continua of care available in the public domain. Search inclusive of results as of November 30, 2016.

We used standard methods to search the public domain for the latest complete or near-complete national care continua (Fig 1). We used the national continua and accompanying methods to evaluate progress towards achieving the 90-90-90 target. In October 2016, we searched PubMed, UNAIDS and WHO reports, national surveillance and program reports, PEPFAR 2016 Country Operational Plans, and conference presentations and/or abstracts for national HIV continua of care for the period 2010–2016. The Google-based search strategy included the keywords (HIV OR AIDS) AND (treatment) AND (cascade OR continuum of care OR care continuum OR continua OR spectrum of care OR 90-90-90 OR viral suppression) for data published in the public domain. Although the search terms were in English and most of the sources were in English, some of the UNAIDS and/or country reports were in French or Spanish. We also searched the following conferences: International AIDS Society (IAS) Conference on HIV Science of 2015 and 2016, the Conference on Retroviruses and Opportunistic Infections of 2015 and of 2016, and the IAPAC Treatment as Prevention and Pre-exposure Prophylaxis Evidence Summit. We also included the following online databases: http://www.unaids.org/en/dataanalysis/knowyourresponse/countryprogressreports/2016countries/ and https://www.pepfar.gov/reports/guidance/250167.htm.

The search was designed to identify the most recent officially reported or sanctioned national continua of care available in the public domain. To explore open data principles, the search was designed to be replicable by most nonacademic researchers, including people living with HIV, community organizations, clinicians, and other people.

From the continua, we collected data on the following four key steps in the treatment cascade: (1) estimated number of people living with HIV; (2) estimated number of people living with HIV diagnosed as HIV positive; (3) estimated number of people living with HIV receiving ART; and (4) estimated number of people living with HIV with a suppressed or undetectable viral load [25,26]. We used the estimated number of people living with HIV as the denominator to calculate the indicators for the continua (e.g., number of people on ART/estimated number of people living with HIV) and the numerators to calculate the 90-90-90 target. We relied on the country report of viral suppression using its lowest quantifiable threshold criteria. If data on the estimated number of people living with HIV (PLHIV) were unavailable, we used UNAIDS estimates to complete the cascade. We reviewed progress towards achieving the UNAIDS 90-90-90 target, which translates into 90% of PLHIV diagnosed, 81% of PLHIV on ART, resulting in 73% of PLHIV with viral suppression.

For countries with continua with viral suppression estimates, we also reviewed and ranked the methods for determining the four key care indicators. We contacted the authors if the care continua did not include methods for determining the “HIV diagnosis,” “on ART,” and “viral suppression” indicators. Using available WHO and International Association of Providers for AIDS Care (IAPAC) guidelines as the standard [25,26], the individual indicators were graded as high-, medium-, or low-quality based on quality of data, methods, and national representativeness (S1 Table key). Medium- and high-quality indicator data are interpreted as “satisfactory” for understanding the care continuum and monitoring the response. The quality of the indicator data was applied to the care continua; they were accorded an overall grade based on the lowest indicator grade.

There are significant challenges involved in the collection of the data necessary to put together a national care continuum. Specifically, without a cohort approach and patient identifiers, it is very challenging to link individual HIV diagnosis with care and treatment outcomes as it is often done in a different location with distinct data collection systems. Additionally, most countries still do not offer immediate treatment, and HIV diagnosis may be separated from starting treatment by many months to years [27]. Likewise, viral load testing is often unavailable for all patients, and laboratory data systems may have difficulty deduplicating multiple samples for the same patient. Although there are an increasing number of high-quality population-based surveys with biologic sampling, the majority of countries rely on antenatal clinic surveillance and complex modelling to derive incidence and prevalence estimates that require wide confidence intervals. Data on HIV diagnosis, on ART, and viral suppression collected directly from program service delivery were accorded the highest value as they are drawn from individuals living with HIV who are receiving services and reflect progress towards targets. Program service delivery data were also classified as the highest grade since they serve as direct feedback regarding program performance. Other data sources such as community-based surveys, select quality assurance efforts, and/or laboratory information are also valuable and may be used to triangulate on the service delivery data—if triangulation using surveillance data was described as part of the methodology, the indicator was upgraded accordingly. High-grade continuum used methodologies that included all of the following: (1) national and/or UNAIDS estimates for the overall denominator of persons living with HIV or similar estimates derived from nationally representative surveys and surveillance, (2) a cohort or national program service database that included everyone diagnosed with HIV, (3) a national cohort or program service database for those on ART, and (4) individual viral load data for everyone on ART or cohort/surveys representative of everyone on ART. A medium grade was given to continuum that used one or more of the following: (1) estimates based on subnational/subpopulation data for the overall denominator, (2) HIV diagnosis based on population-based surveillance estimates (i.e., not program data) and/or diagnoses in select subpopulations, (3) ART coverage estimates using subpopulation cohorts or population-based surveillance (i.e., not program data), and/or (4) viral load suppression measured from a subsample of those on ART or population-based surveillance surveys (i.e., not program data). The medium grade was attributed to methodologies that used a population-based surveillance study or system since they can provide an accurate estimate of steps in the care continuum including viral suppression but do not directly measure program service delivery. Additionally, most surveys rely on self-report for being on ART. A low grade was given to continuum with the following characteristics: (1) estimates from modeling studies, (2) diagnosis derived from data in a nonrepresentative selection of clinics and/or hospitals or the numerator was not calculated, (3) ART coverage based on a nonrepresentative selection of clinics and/or hospitals, and/or (4) viral load data from a nonrepresentative selection of clinics and/or hospitals. If the source of one indicator was unknown, the continuum was ranked according to the grade of the remaining three indicators. The quality of the continuum was labeled as “unknown” if sources of the three indicators—i.e., PLHIV diagnosed, on ART, and with viral suppression—were unknown. This article was cleared by both Centers for Disease Control and Prevention and IAPAC. All data used for the study are available in the public domain at www.HIV90-90-90watch.org.

## Results

The PubMed search located 1,482 studies, which yielded 34 reported national care continua or cascades for all PLHIV (Fig 1). The wider Internet search located 127 reported national care continua. In case of multiple continua for an individual country, we used the most recently reported care continua to reduce the eligible number of care continua from 161 to 97. Of the 97, we excluded 15 care continua that were from secondhand reports and/or reported as preliminary and unconfirmed national figures. Of the 82 remaining care continua, we abstracted data on the four indicators. For the three countries missing estimates of PLHIV, we completed this portion of the care continuum using UNAIDS estimates [28,29]. The 29 continua that did not have data to calculate viral load suppression among PLHIV were excluded from further analysis and quality assessment. National care continua from 53 countries with viral suppression estimates were analyzed in detail. Of these, 9 care continua did not include the methods for determining the HIV diagnosis, on ART, and viral suppression indicators. Of these, we contacted the authors and obtained the methods for 2 continua.

We found care continua from 82 countries in the public domain from 2010–2016, representing 33.8 million (92%) of the 2015 global estimate of PLHIV [28]. Of the 82 countries, only Sweden has achieved the 90-90-90 target (Table 1). Data on PLHIV diagnosed were available for 51 of 82 countries, data for the on ART indicator for 80 of 82 countries, and data for viral suppression for 53 of 82 countries. National care continua from these 53 countries are analyzed below.

**Table 1:** Number and proportion of people living with HIV, diagnosed, on ART, and with viral suppression for 82 countries (2010–2016).

NO.	COUNTRY	YEAR	ART eligibility criteria (CD4 cells/mm^3^)	People living with HIV		People living with HIV diagnosed		People living with HIV receiving ART			People living with HIV with suppressed/undetectable viral load		
				Number^a^	% of PLHIV	Number	% of PLHIV	Number	% of PLHIV	% of PLHIV diagnosed	Number	% of PLHIV	% of PLHIV on ART^b^
**COUNTRIES WITH CONTINUA WITH VIRAL SUPPRESSION ESTIMATES**													
1	Argentina	2013	<500	110,000	100%	78,400	71%	52,034	47%	66%	34,342	31%	66%
2	Armenia	2013	<350*	3,503	100%	1,310	37%	579	17%	44%	510	15%	88%
3	Australia	2013	All	26,800	100%	23,100	86%	17,700	66%	77%	16,600	62%	94%
4	Belarus	2014	<200	25,000	100%	15,700	63%	5,181	21%	33%	3,150	13%	61%
5	Belgium	2011	All*	20,000^a^	100%	12,639	63%	10,532	53%	83%	8,784	44%	83%
6	Brazil	2015	All	827,000	100%	715,000	86%	455,000	55%	64%	410,000	50%	90%
7	Cambodia	2015	<500	72,607	100%	60,336	83%	54,769	75%	91%	50,935	70%	93%
8	China	2014	<500	825,000	100%	501,000	61%	295,358	36%	59%	267,447	32%	91%
9	Colombia	2013	<500	122,953	100%	55,329	45%	40,739	33%	74%	28,279	23%	69%
10	Cuba	2013	<200	19,626	100%	14,648	75%	8,102	41%	55%	5,898	30%	73%
11	Denmark	2010	<350	5,800^a^	100%	5,519	95%	4,029	69%	73%	3,863	67%	96%
12	El Salvador	2014	<500	20,874	100%	14,403	69%	6,471	31%	45%	4,592	22%	71%
13	Estonia	2013	<200	9,200	100%	8,004	87%	2,691	29%	34%	1,748	19%	65%
14	France	2010	<350	148,900	100%	119,900	81%	90,000	60%	75%	76,900	52%	85%
15	Georgia	2015	All*	7,100	100%	4,339	61%	2,685	38%	62%	2,274	32%	85%
16	Guatemala	2014	<350	47,800	100%	28,537	60%	16,965	35%	59%	8,747	18%	52%
17	Guyana	2014	<350	9,665	100%	N/A		4,295	44%		3,007	31%	70%
18	Honduras	2014	<500	23,000	100%	12,167	53%	9,752	42%	80%	7,935	35%	81%
19	Jamaica	2014	<350	29,690	100%	23,915	81%	8,781	30%	37%	6,459	22%	74%
20	Kazakhstan (15+)	2015	<350	19,838	100%	18,185	92%	6,031	30%	33%	3,377	17%	56%
21	Kenya	2015	<500	1,366,771	100%	N/A		857,472	63%		726,370	53%	85%
22	Kyrgyzstan (15+)	2015	<350*	8,865	100%	N/A		1,470	17%		897	10%	61%
23	Lao People’s Democratic Republic	2014	<200	11,556	100%	4,730	41%	2,787	24%	59%	1,954	17%	70%
24	Malawi	2015	<500	1,065,491	100%	N/A		613,227	58%		521,243	49%	85%
25	Malaysia	2014	<350	100,000	100%	N/A		21,654	22%		19,922	20%	92%
26	Mauritius	2014	<350*	9,200	100%	5,137	56%	3,206	35%	62%	2,001	22%	62%
27	Mexico	2014	All	190,000	100%	119,200	63%	98,000	52%	82%	64,000	34%	65%
28	Mongolia	2014	N/A	772	100%	139	18%	126	16%	91%	111	14%	88%
29	Myanmar	2014	<500	212,560	100%	N/A		85,626	40%		74,495	35%	87%
30	Namibia	2016	<500	229,631	100%	N/A		161,785	70%		143,989	63%	89%
31	Nepal	2015	<500	39,397	100%	22,267	57%	11,922	30%	54%	10,730	27%	90%
32	Netherlands	2014	All	25,000	100%	19,065	76%	16,081	64%	84%	14,602	58%	91%
33	Nicaragua	2014	<350	10,036	100%	7,760	77%	2,935	29%	38%	1,761	18%	60%
34	Panama	2014	<350	16,565	100%	13,583	82%	8,283	50%	61%	5,301	32%	64%
35	Paraguay	2014	<350	16,825	100%	12,388	74%	4,707	28%	38%	3,285	20%	70%
36	Philippines	2014	<200	36,000	100%	22,527	63%	8,481	24%	38%	7,548	21%	89%
37	Romania	2014	All	14,000	100%	12,886	92%	9,571	68%	74%	5,148	37%	54%
38	Russia	2013	<500	1,363,330	100%	668,032	49%	156,858	12%	23%	127,054	9%	81%
39	Rwanda	2015	<500	212,642	100%	N/A		156,471	74%		136,292	64%	87%
40	South Africa	2015	<500	6,669,360	100%	N/A		3,217,097	48%		2,541,507	38%	79%
41	Spain	2013	<500	150,000	100%	106,500	71%	73,881	49%	69%	68,709	46%	93%
42	Sri Lanka	2014	<500	3,600	100%	1,737	48%	644	18%	37%	299	8%	46%
43	Suriname	2012	<200*	3,800^a^	100%	3,274	86%	735	19%	22%	478	13%	65%
44	Swaziland	2016	<500	226,920	100%	151,287	67%	144,412	64%	95%	122,750	54%	85%
45	Sweden	2015	<350	7,718	100%	6,946	90%	6,605	86%	95%	6,053	78%	92%
46	Switzerland	2012	<350	15,800	100%	12,800	81%	11,200	71%	88%	10,700	68%	96%
47	Tajikistan (15+)	2015	<350*	15,280	100%	5,310	35%	2,323	15%	44%	1,951	13%	84%
48	Thailand	2014	All	445,642	100%	356,514	80%	271,652	61%	76%	260,786	59%	96%
49	Uganda	2014	<500	1,500,000	100%	N/A		750,896	50%		675,806	45%	90%
50	Ukraine	2015	<500	220,000	100%	126,604	58%	68,455	31%	54%	53,190	24%	78%
51	United Kingdom	2014	<350	103,700	100%	85,600	83%	76,900	74%	90%	72,800	70%	95%
52	United States	2012	All	1,218,400	100%	1,062,100	87%	441,422	36%	42%	368,338	30%	83%
53	Zimbabwe	2015	<500	1,400,000	100%	N/A		879,271	63%		723,640	52%	82%
**COUNTRIES WITH CONTINUA WITHOUT VIRAL SUPPRESSION ESTIMATES**													
1	Angola	2014	<350	300,000	100%	N/A		68,507	23%		N/A		
2	Antigua and Barbuda	2014	<500*	N/A		750		254		34%	N/A		
3	Bahamas	2014	<350*	6,979	100%			2,212	32%				
4	Bangladesh	2014	<500	8,935	100%	3,111	35%	1,287	14%	41%	N/A		
5	Barbados	2013	<350*	2,147	100%			1,043	49%		571		
6	Bhutan	2014	<500	1,000	100%	403	40%	167	17%	41%	N/A		
7	Botswana	2015	<350	392,435	100%	N/A		273,904	70%		N/A		
8	Burundi	2015	<500	84,000	100%	N/A		42,169	50%		N/A		
9	Cameroon	2015	<500	683,387	100%	N/A		168,249	25%		N/A		
10	Cote d'Ivoire	2015	<350	460,000	100%	195,755	43%	147,947	32%	76%	N/A		
11	Dominican Republic	2015	<500*	67,544	100%	N/A		32,291	48%		N/A		
12	Egypt	2014	<350*	7,200	100%	4,631	64%	1,715	24%	37%	N/A		
13	Ethiopia	2015	<500	729,515	100%	N/A		373,933	51%		N/A		
14	Ghana	2015	<350	270,000	100%	N/A		89,113	33%		N/A		
15	Haiti	2015	<500	141,269	100%	N/A		66,528	47%		N/A		
16	India	2014	<350	2,100,000	100%	1,400,000	67%	747,175	36%	53%	N/A		
17	Indonesia	2015	<350	690,000	100%	200,618	29%	63,066	9%	31%	N/A		
18	Lesotho	2015	<500	315,002	100%	N/A		146,790	47%		N/A		
19	Morocco	2013	<350	N/A		4,032		3,620		90%	N/A		
20	Mozambique	2016	All	1,643,065	100%	N/A		802,659	49%		N/A		
21	Nigeria	2014	<350	3,413,245	100%	929,995	27%	743,996	22%	80%	N/A		
22	Papua New Guinea	2015	<350	40,148	100%	N/A		21,198	53%		N/A		
23	Somalia	2013	<350*	26,350	100%	5,581	21%	1,700	6%	30%	N/A		
24	South Sudan	2015	<500	193,376	100%	N/A		15,715	8%		N/A		
25	Tanzania	2014	<500	1,400,000	100%	N/A		740,078	53%		N/A		
26	Timor-Leste	2014	<350	464	100%	N/A		173	37%		N/A		
27	Trinidad	2014	<500*	13,000	100%	N/A		6,399	49%		N/A		
28	Vietnam	2015	<500	260,236	100%	202,437	78%	106,423	41%	53%	N/A		
29	Zambia	2016	<500	1,151,201	100%	N/A		721,016	63%		N/A		

Abbreviations: ART, antiretroviral therapy; HIV, human immunodeficiency virus; N/A, data not available; PLHIV, people living with HIV. These data are available at www.hiv90-90-90watch.org. The percentage was multiplied by the number of people receiving ART to obtain the number of people on ART estimated to be virally suppressed. Note: The percentages in green show the countries that have achieved one or more of the UNAIDS 90-90-90 target.

^a^ UNAIDS estimates.

^b^ For many countries, the number represents the “percentage of people on ART tested for viral load who have a suppressed viral load in the reporting period.”^.^

* Reported ART eligibility criteria. For rest of the countries, the ART eligibility criteria are from the published national guidelines.

The 53 national care continua with viral suppression estimates represented 19.7 million (54%) of the 2015 global estimate of PLHIV [28]. The continua included 13 from European countries, 15 from North and South America, 15 from Asia, 9 from Africa, and 1 from Australia (Table 2). Care continua with viral suppression estimates were available in the public domain for only nine countries in sub-Saharan Africa (Kenya, Malawi, Mauritius, Namibia, Rwanda, South Africa, Swaziland, Uganda, and Zimbabwe), representing 35% of the 2015 HIV burden.

**Table 2:** Number and proportion of people living with HIV, diagnosed, on ART, and with viral suppression for 53 countries with viral suppression estimates: Stratified by quality, region, and results (2010–2016).

		Number of countries	Global HIV burden in 2015	People living with HIV (PLHIV)	People living with diagnosed HIV	Proportion of PLHIV diagnosed (range)	People receiving ART	Proportion of PLHIV receiving ART (range)	People on ART with suppressed viral load	Proportion of PLHIV with suppressed viral load (range)
	**ALL CONTINUA**	**53**	**54%**	**19,283,217**	**N/A**	**NA (18%–95%)**	**9,257,849**	**48% (12%–86%)**	**7,718,557**	**40% (8%–78%)**
**By Quality**	High	6	2%	890,618	549,508	62% (49%–95%)	335,290	38% (36%–86%)	303,023	34% (32%–78%)
	Medium	28	15%	5,304,575	3,772,733	71% (18%–92%)	2,031,991	38% (12%–75%)	1,734,084	33% (8%–70%)
	Low	14	36%	12,965,785	N/A	N/A (63%–81%)	6,839,526	53% (17%–71%)	5,649,727	44% (10%–68%)
	Unknown	5	<1%	122,239	81,576	67% (53%–92%)	51,042	42% (31%–68%)	31,723	26% (18%–37%)
**By Region**	Africa	9	35%	12,680,015	N/A	N/A (56%–67%)	6,783,837	54% (35%–74%)	5,593,597	44% (22%–64%)
	Americas	15	7%	2,666,234	2,160,704	81% (45%–87%)	1,158,221	43% (19%–55%)	952,422	36% (13%–50%)
	Asia	15	5%	1,801,720	N/A	N/A (18%–92%)	766,107	43% (15%–75%)	703,236	39% (8%–70%)
	Australia	1	<1%	26,800	23,100	86%	17,700	66%	16,600	62%
	Europe	13	6%	2,108,448	1,200,195	57% (49%–95%)	531,984	25% (12%–86%)	452,701	21% (9%–78%)
**Top 10 Countries**	Sweden*	1	<0.1%	7,718	6,946	90%	6,605	86%	6,053	78%
	Cambodia**	1	0.2%	72,607	60,336	83%	54,769	75%	50,935	70%
	United Kingdom**	1	0.3%	103,700	85,600	83%	76,900	74%	72,800	70%
	Switzerland***	1	<0.1%	15,800	12,800	81%	11,200	71%	10,700	68%
	Denmark*	1	<0.1%	5,800	5,519	95%	4,029	69%	3,863	67%
	Rwanda***	1	0.5%	212,642	N/A	N/A	156,471	74%	136,292	64%
	Namibia***	1	0.6%	229,631	N/A	N/A	161,785	70%	143,989	63%
	Australia**	1	0.1%	26,800	23,100	86%	17,700	66%	16,600	62%
	Thailand**	1	1.2%	445,642	356,514	80%	271,652	61%	260,786	59%
	Netherlands*	1	0.1%	25,000	19,065	76%	16,081	64%	14,602	58%

Abbreviations: N/A, not available. Data for the indicators PLHIV, diagnosed, on ART, and with viral suppression have been aggregated over varying years. Note: Green denotes 90% or more PLHIV diagnosed, 81% or more PLHIV on ART, or 73% or more PLHIV with viral suppression. Yellow denotes 70%–89% PLHIV diagnosed, 60%–80% PLHIV on ART, or 50%–72% PLHIV with viral suppression. Red denotes <70% PLHIV diagnosed, <60% PLHIV on ART, or <50% PLHIV with viral suppression.

* denotes high-quality continuum

** denotes medium-quality continuum

*** denotes low-quality continuum.

**Fig 2:**
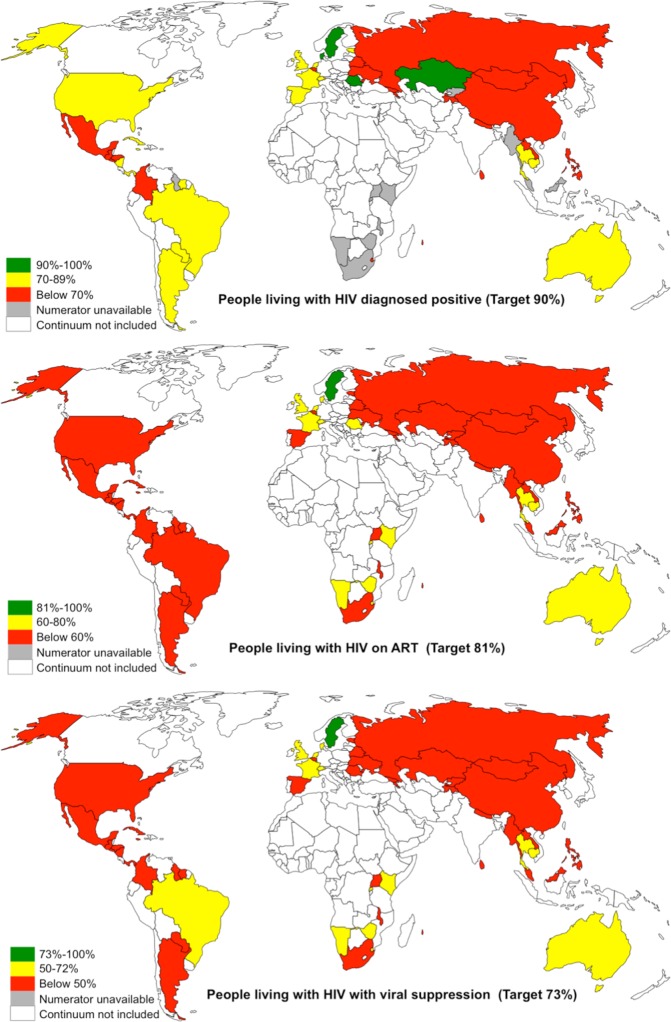
Maps showing the proportion of PLHIV diagnosed positive, on ART, and with viral suppression in 53 countries. Countries in white were not included in this analysis (see methods for exclusion criteria). Maps are available from www.freeworldmaps.net.

**Fig 3:**
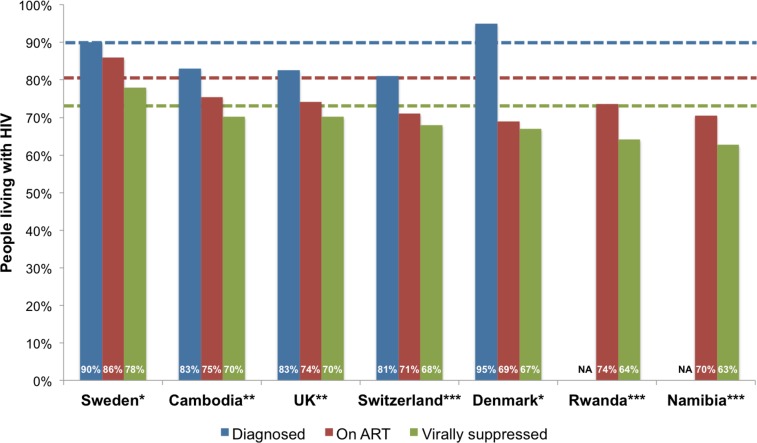
Top seven countries within 12% and 10% of achieving the 90-90-90 target for on ART and for viral suppression, respectively. Note: the blue line denotes the first 90 target of 90% of PLHIV diagnosed, the red line denotes the second 90 target of 81% of PLHIV on ART, and the green line denotes the third 90 target of 73% PLHIV with viral suppression. * denotes high-quality continuum, ** denotes medium-quality continuum, *** denotes low-quality continuum.

Of the 53 countries, 4 (Denmark, Kazakhstan, Romania, and Sweden) achieved the first 90 target of 90% PLHIV diagnosed, one (Sweden) achieved the second 90 target of 81% on ART, and one (Sweden) achieved the third target of 73% viral suppression (Table 1 and Fig 2). The proportion of PLHIV diagnosed on ART was >90% in 5 countries, and viral suppression among PLHIV on ART tested for viral load was >90% in 14 countries (Table 1). Seven countries (Sweden, Cambodia, UK, Switzerland, Denmark, Rwanda, and Namibia) have reached or were within 12% and 10% of achieving the 90-90-90 target for on ART and for viral suppression, respectively (Fig 3). Of the 53 countries, the percentage of PLHIV diagnosed was between 70%–89% in 18 countries, ART coverage among PLHIV was between 60%–80% in 14 countries, and viral suppression was between 50%–72% in 14 countries (Fig 2). The 53 countries averaged 48% of PLHIV on ART (the denominator was the aggregate of PLHIV in 53 countries) and 40% for viral suppression (Table 2). The average was not calculated for PLHIV diagnosed since many countries were missing data on this indicator. When restricted to those with care continuum from 2014 onwards (39 countries), these numbers were 52% and 43%, respectively. The 9 sub-Saharan African countries averaged 54% of PLHIV on ART and 44% for viral suppression (the denominator was the aggregate of PLHIV in 9 countries).

**Fig 4:**
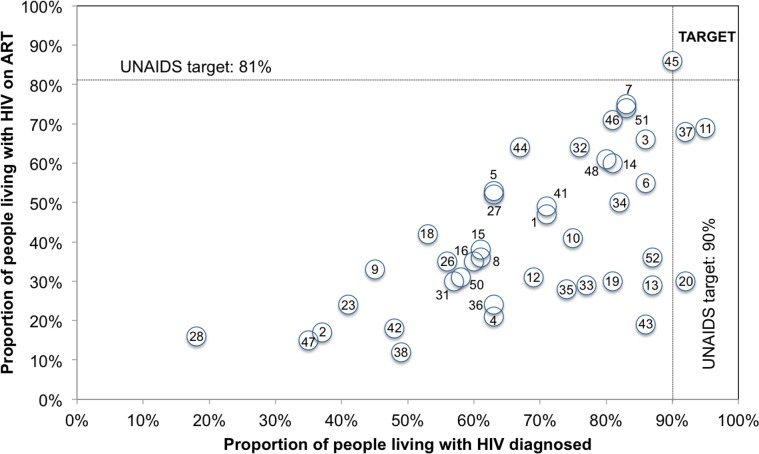
Country progress toward the 90-90-90 target: Proportion of PLHIV diagnosed and on ART for 53 countries. Note: the figure includes 53 countries. The numbers in the figure above correspond to the number for the countries with continuum with viral suppression as listed in Table 1. The proportion of PLHIV with diagnosed infection was not available for 11 countries. Note that this graph is meant to illustrate progress and does not calculate correlation between the variables as they are not independent.

**Fig 5:**
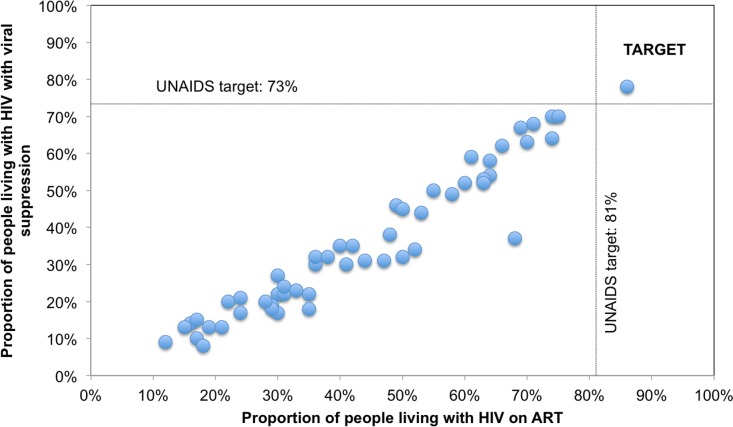
Country progress toward 90-90-90 target: Proportion of PLHIV on ART and with viral suppression for 53 countries. The data points in the figure were not labeled with numbers, as they are clustered and overlapping. Note that this graph does not show correlation between the variables as they are not independently determined—the graph is only meant to portray progress for the two variables towards the target.

Progress towards the 90-90-90 target varied by country (Figs 4 and 5). The proportion of PLHIV on ART was generally higher in countries with a higher proportion of PLHIV diagnosed (Fig 4). However, countries with higher proportions of PLHIV diagnosed had varying proportions of ART coverage (19%–86%). Some countries are close to achieving the second and third 90-90-90 target; however, there is a range of estimated viral suppression (Fig 5).

Of the 53 continua, 6 (2% of the global HIV burden) were high quality, using standard surveillance methods to derive an overall denominator and program data from national cohorts for PLHIV with an HIV diagnosis, on ART, and with viral suppression (S1 Table and S2 Table—source of information and grading of methods quality). There were 28 (15% HIV burden) of medium quality, using only estimates for number of people diagnosed and/or on ART and/or viral load suppression. Fourteen continua (36% burden) were of low quality, and 5 (<1% burden) were unable to be graded because of missing data.

## Discussion

Estimating national HIV continuum of care helps to guide the global and local HIV response, and our study found that many national HIV programs have published their continuum for everyone living with HIV [9,10,13,14,21–23]. Recognizing the importance of expanding treatment, international donors, including PEPFAR and GFATM, are supporting efforts to achieve the 90-90-90 target and care continua to improve accountability and ensure impact [30]. Our findings suggest that the substantial investment in expanding access to HIV diagnosis and treatment has resulted in significant progress towards the 90-90-90 target. The review also highlights that despite this investment, there is a lack of complete data available in the public domain coupled with a nonstandardized approach to determining national continua. The missing data and lack of standardization makes it difficult to determine with confidence whether many countries are on track to achieve the 90-90-90 target. However, although some of the program care continua data may be unavailable or unclear, recent population-based studies suggest that HIV program performance may be better than reported and treatment expansion has had a significant impact on viral suppression and HIV incidence [31].

### Progress toward the 90-90-90 target

Complete information regarding national care continuum is difficult to find in the public domain, and we found only 53 national continua representing 54% of global HIV burden with viral suppression estimates (some of these were missing estimates for PLHIV diagnosed). Despite having 70% of the global burden for HIV in 2015 and 47% of the reported low- and middle-income country investment in the HIV response in 2012 [28,32], only nine countries from sub-Saharan Africa had continua in the public domain with viral suppression estimates. This lack of comprehensive data may be partly explained by obsolete “test and wait” strategies based on previous WHO guidelines. These strategies often include a fragmented monitoring and evaluation system resulting from stand-alone testing services combined with referral and delayed treatment only when a patient is very ill or when s/he is determined by CD4 count measurement to be severely immunocompromised. An additional obstacle is that measuring viral load suppression is a relatively new component of the WHO recommended treatment strategy and access for people taking ART has been delayed in most settings. Alternatively, the missing care continua may not be in the public domain and may only be available to experts in Ministries of Health or other agencies. Perhaps the information is available using more sophisticated search methods or through reconstruction of continua using a variety of surrogate sources. Regardless, the lack of easily available continua information, including trends, for most countries suggests that there are still significant obstacles preventing both the estimation and publication of progress towards the 90-90-90 target.

The results indicate that Sweden has achieved the 90-90-90 target, and a number of countries are close to achieving the first and second 90 targets by 2020. However, global progress towards achieving the 90-90-90 target and epidemic control relies on achieving the third and most important target of 73% viral suppression. Of the 53 countries with data on PLHIV with viral suppression, only 15 (14% of 2015 global burden) reported >50% viral suppression levels. This has serious implications and may reflect lower proportions of people diagnosed with HIV and on ART or that many countries are still not benefiting from the science that clearly supports the health and prevention benefits from initiating ART irrespective of CD4 cell count [3–6,33–36]. In September 2015, WHO issued global guidelines reflecting the scientific data that indicate health and transmission benefits from initiating ART irrespective of CD4 cell count [37]. However, although 33 countries have published guidelines recommending “test and treat,” the majority of the 53 countries in our review still follow the now obsolete CD4 <200, <350, or <500 cells/mm^3^ ART initiation criteria [27]. The review suggests that countries that have published guidelines promoting earlier access to treatment including “test and treat” reported continua with an increased proportion of PLHIV who have a suppressed viral load.

### Evaluating continua quality

For the 48 of the 53 continua with available methods, there was a considerable lack of standardization and quality. Of the available continua, only six countries with 2% of global HIV burden could be classified as having the highest-quality methods conforming to international recommendations [25,26]. Although grading continua includes a degree of subjective judgment, the task was complicated since many reported continua presented data without describing the methods used to estimate and calculate the individual indicators. Care continua for key populations, tuberculosis, or other groups are even less available in the public domain and often rely on lower-quality estimation methods. Our study illustrates that the lack of standardized and quality methods impacts interpretation of the results for all of the 90-90-90 targets across countries. Comparison of national progress requires caution and should be accompanied by a critical review of not only the reported results but also the methods used to derive the care continua indicators.

### Challenges determining progress toward the 90-90-90 target

Estimating the First 90 was plagued by a lack of consistency in the methods used to determine HIV prevalence and the proportion of people diagnosed with HIV. Reliable prevalence estimates require population-based HIV surveillance or surveys [38] that include biologic data such as HIV diagnosis, viral load, presence of antiretrovirals (ARVs), and CD4 cell count combined with measurement of the impact of program implementation on outcomes such as HIV incidence and mortality. Although higher-quality estimates are feasible and have been made in some countries [38], most countries reviewed depended on modeled UNAIDS or national estimates using antenatal clinic or other subpopulation data or national consensus regarding the number of PLHIV. The recently completed population-based survey in three African countries illustrates the potential problems with modeling as it found incidence estimates in Zimbabwe that were around half of those reported by UNAIDS [31]. For estimating the number of people diagnosed with HIV, most countries rely on aggregate data from HIV testing efforts, estimates of those in care, and/or periodic surveys with self-reporting of known status with varying success, timeliness, and confidence. Reporting on the proportion of people with an HIV diagnosis is often on the basis of the number of tests done and the number found positive, which, without identifiers, makes it difficult to determine the number of new individuals tested and the overall proportion of HIV cases detected. Additionally, since HIV testing is often carried out in both governmental and nongovernmental settings, it is often impossible to say with certainty whether those with a positive HIV test access treatment. Without individual identifiers or other means to count newly diagnosed individual persons, there is no way to ensure linkage to care or record deaths, and data therefore may include an unknown amount of retesting by persons previously diagnosed. The clear benefits of immediate treatment for the prevention of illness, death, and transmission highlight the importance of establishing a monitoring and evaluation system that is accountable for everyone who receives an HIV diagnosis so that they can access treatment and achieve viral suppression.

Reporting for the Second 90 was available for most countries included in the review that used national program service data to report the number and proportion of people on ART. However, most did not describe what criteria are used to define “receiving or on ART” or other efforts to insure data quality, including deduplication to avoid overcounting. The IAPAC Continuum of Care Guidelines recommend using a defined program measure such as at least one drug pickup during the measurement period [26]. WHO guidance recommends that ART patients seen within 90 d of a last drug pickup appointment, and excluding those who are deceased, are considered to be receiving ART [25]. While ensuring access to treatment is critical, it is also important to be able to monitor and evaluate long-term retention in care and viral suppression. However, given the challenges posed by adherence and retention and the variance in how it is defined, the number of people reported “receiving or on ART” may be an unreliable measure of the actual number of people who are actually virally suppressed. Directly measuring viral suppression for everyone on ART is the best means to determine whether a person is both receiving and taking ART.

Measuring the Third 90 is challenging for many countries, and only nine countries reported directly measuring viral load for all of the people on ART. The majority relied on the viral suppression rate among samples at the national laboratory to infer the proportion of people on ART who are suppressed. If most people on ART routinely receive a viral load test within the reporting period and duplication is avoided, then this can give a reasonable approximation of the proportion of people who are suppressed. However, in most settings viral load tests are not standard, may be duplicated, and are instead used in the context of assessing patients for failure due to drug resistance or nonadherence. In this situation, the level of suppression among everyone on ART may actually be much higher than reported for only those who are potentially failing; however, if many people who do not have viral load measures are no longer in care, then using unadjusted data from a national laboratory subsample of those in care could actually overestimate viral suppression. As viral load testing becomes increasingly available, it will be possible to move to direct measurement of viral suppression among people on ART, through routine viral load monitoring, as the best measure of program success.

### Limitations

Our study has some limitations. In an effort to indirectly measure accountability and progress, we purposefully relied on official national care continua in the public domain. Therefore, our exhaustive public domain search may have missed other continua that have been recently published or are available only to health authorities or researchers. Additional challenges for accurately reporting comprehensive national care continua include siloed data collection with separate testing and treatment services, obsolete “test and wait” treatment protocols, lack of individual patient identifiers, and inability or unwillingness to adopt a unified single national cohort approach. More accurate and higher-quality continua measurement could be obtained by removing duplicate and redundant data collection efforts along with the adoption of unique identifiers and a standardized unified national cohort approach applied across all HIV services to help ensure the linkage of individuals to successful ART and viral suppression results. The study represents a single point in time, many countries do not regularly update published information, and we relied on the latest published or presented information, sometimes as old as from 2010. Additionally, while most continua described their methodologies, the quality of data collection and other more detailed methodological issues may have been missed. The heterogeneity in country-specific methods for monitoring the continua of care creates complex issues when comparing progress towards targets across countries. Only a few countries could produce national cohorts and/or national program data that include HIV diagnosis through viral suppression. Most countries reported derived estimates of continua numerators, which may be prone to data errors and methodological flaws. Specifically, without patient identifiers and a cohort system, patient loss after diagnosis, duplication of patients in the system, and lack of individually linked viral load results may compromise the validity of some continua. Also, this study did not evaluate the availability of high-burden subnational or population-specific care continuums (e.g., key populations), which may more directly inform strategies for targeted program improvement and community incidence interruption [39].

### Recommendations

Despite ongoing efforts and considerable investments to improve monitoring and evaluation, our review highlights the need for improved standardization across countries to accurately track progress towards epidemic control. Guidelines for measuring continuum indicators exist [25,26]. To ensure accountability and measure progress towards 90-90-90, and there needs to be a renewed commitment by national governments and international donors to use a standardized continuum methodology. Refocusing investment to support standardization of national HIV cascade monitoring and evaluation according to the IAPAC Guidelines for Optimizing the HIV Care Continuum for Adults and Adolescents and the WHO Consolidated Strategic Information guidance may help to improve availability and quality of care continua [25,26]. As part of this effort, it will be important to clearly define and describe standard numerators and denominators so that “diagnosed HIV,” “receiving ART,” “on ART,” and “viral suppression” have the same meaning regardless of national setting. Additionally, continuum for patients with HIV-associated tuberculosis, pregnant women, youth, and key populations may also prove to be useful [39]. While accurate program data are essential to monitor service delivery and progress, routine case-based surveillance can also be considered as a tool to monitor generalized HIV epidemics or infections in key populations as well as to promote linkage to care [40]. Resources devoted to testing and treatment services, strategic information, health systems strengthening, and community support should be prioritized to focus on building standardized national cohorts with individual patient identifiers and the ability to easily produce accurate national and subnational care continua. Periodic population-based household-level surveys that include biologic sampling for HIV, viral load, CD4 cell counts, and ARV drug levels should also be included as a means to corroborate program data and monitor progress towards ending AIDS.

Our study suggests that there is also considerable room for innovation in reporting progress to 90-90-90 and epidemic control. Our search revealed that essential data are all too often “buried” in inflexible, increasingly obsolete PDF formats, segmented or too old to meaningfully inform decision making. There is considerable potential for cloud-based software platforms that can provide multiple data visualization options and near real-time reporting of progress towards the 90-90-90 target and other important outcomes. Near real-time national and subnational monitoring could greatly improve the capacity to evaluate program implementation and thus further improve service delivery. Internet-based reporting using open data principles while protecting confidentiality could provide open access for health experts and the community, with the potential benefit of improving transparency, accountability, and timeliness of reporting. Without a significant improvement in quality and availability of data, it will be very challenging to successfully match adequate resources with gaps in progress towards the 90-90-90 target.

### Conclusion

Decisions regarding monitoring and evaluation methodologies have major strategic implications. While it is clear that many countries are progressing toward and will likely achieve 90-90-90, it will be important to use consistent and accurate methods to report on progress. Specifically, the lack of standardization and variable quality suggest that reports of national continua and progress towards 90-90-90 require careful scrutiny [7]. The benefits of standardized and comparable continua include improved accountability and stewardship of the scarce international and national program resources directed towards 90-90-90 and epidemic control. Keeping the important issue of individual patient confidentiality in mind, a standardized continuum system based on a unique identifier and a national program cohort of everyone living with diagnosed HIV would be a major step towards ensuring that everyone living with HIV has access to HIV diagnosis and treatment. Publishing high-quality continua of care data in the public domain could improve efficiency, transparency, and accountability and is essential to focus scarce resources on achieving 90-90-90 and epidemic control.

## Supporting information

S1 PRISMA Checklist PRISMA checklist. (DOC)

S1 Table Summary and grading of the quality of sources of information on the four key steps in the HIV continua of care for 53 countries that have estimates on viral suppression. (DOCX)

S2 Table Summary and grading of the sources of information on the four key steps in the HIV continua of care for 29 countries that do not have estimates on viral suppression. (DOCX)

## Abbreviations

ART: antiretroviral treatment
ARV: antiretroviral
GFATM: the Global Fund Against AIDS, Tuberculosis and Malaria
HIV: human immunodeficiency virus
IAPAC: International Association of Providers for AIDS Care
IAS: International AIDS Society
PEPFAR: United States President’s Emergency Plan for AIDS Relief
PLHIV: people living with HIV
UNAIDS: the Joint United Nations Program on HIV/AIDS
USD: United States dollars
WHO: World Health Organization
