# Supplementary material for: Status and methodology of publicly available national HIV care continua and 90-90-90 targets: A systematic review
Source: PLoS Med. 2017 Apr 4;14(4):e1002253. doi: 10.1371/journal.pmed.1002253 (PMC5380306; doi:10.1371/journal.pmed.1002253)
Supplement: S1 Table — (DOCX) [file pmed.1002253.s002.docx]

**Supporting Information**

**Table 1:** Summary and grading of the quality of sources of information on the four key steps in the HIV continua of care for 53 countries, which have estimates on viral suppression.

**KEY**

| **COLOUR** | **Estimated PLHIV** | **PLHIV Diagnosed** | **PLHIV on ART** | **PLHIV with Viral Suppression** |
| --- | --- | --- | --- | --- |
| High quality | - National estimates* - UNAIDS estimate (Spectrum)* - Survey or surveillance data | – National program data  – National cohort data | – National program data  – National cohort data | Individual viral load data for everyone on ART from program database; surveys of patient cohort representative of everyone on ART; nationally-representative population surveys |
| Medium quality | Estimates based on sub-national/sub-population data | Estimates based on surveillance estimates and/or diagnosis in select sub-population | ART coverage estimated using sub-population cohort or surveillance | Estimates based on sub-sample of those on ART or surveillance/ surveys |
| Low quality | - Estimates based on modeling studies  - Numerator is unavailable | Estimates derived from non-representative selection of clinics and/or hospitals | Coverage based on non-representative selection of clinics and/or hospitals | Estimates from non-representative selection of clinics and/or hospitals |
|  | Source and quality unknown | | | |

PLHIV – people living with HIV

| **COUNTRY** | **SOURCE** | **ESTIMATED PEOPLE LIVING WITH HIV** | **PEOPLE LIVNG WITH HIV DIAGNOSED** | **PEOPLE LIVING WITH HIV ON ART** | **PEOPLE LIVING WITH HIV WITH VIRAL SUPPRESSION** | **QUALITY** |
| --- | --- | --- | --- | --- | --- | --- |
| Argentina^1^ | National program report | UNAIDS estimate | Estimated using data on notifications of new HIV infections and deaths | Registers of AIDS Direction (Ministry of Health), registrations in semiprivate and private health subsystems | VL <50 copies/mL Data from AIDS Direction. Calculated using a sample of people on ART receiving VL | Medium |
| Armenia^2^ | UNAIDS meeting report | UNAIDS estimate | Source and quality unknown | National Center for AIDS Prevention (NCAP), Ministry of Health | VL <250 copies/mL Based on data from NCAP laboratory | Medium |
| Australia^3^ | National surveillance report | Diagnosed + Undiagnosed (based on cross-sectional prevalence surveys and on reported HIV and AIDS cases) | National HIV Registry and estimation of deaths | ART coverage is estimated as average of 4 approaches: ARV prescription count (Australian HIV Observational Database or AHOD); self-reported ART use in large national survey; pharmacy dispensing data from New South Wales; study in Victoria analyzing data on ARVs and non-identified individuals receiving ART in Melbourne | VL <400 copies/mL  Calculated as proportion of people with viral suppression recorded in AHOD (cohort size of 3,972) | Medium |
| Belarus^4^ | National program review by WHO | Numbers based on estimate & personal communications with the Infectious Disease Hospital in Minsk | Numbers based on estimate & personal communications with the Infectious Disease Hospital in Minsk | Numbers based on estimate and personal communications with the Infectious Disease Hospital in Minsk | Numbers based on estimate and personal communications with the Infectious Disease Hospital in Minsk | Low |
| Belgium^5,6^ | National cohort data | UNAIDS estimate | National registration of new diagnosis | National cohort data | VL <500 copies/mL National cohort data | High |
| Brazil^7^ | Country presentation | Sistema de Informacao de Agravos de Notificacao or System for notifiable diseases information (SINAN) and Sistema de Informacao de Mortalidade System on Information on Mortality (SIM) | SINAN and SIM | Sistema de Controle Logistico de Medicamentos or Logistics Control System of Medicines (SICLOM) | VL <1,000 copies/mL Sistema de Controle de Exames Laboratoriais or System for Laboratory Tests Control (SISCEL) | Medium |
| Cambodia^8^ | PEPFAR Country Operational Plan | UNAIDS estimate | Calculated as: # of pre-ART + ART patients at end of 2014 plus new positive diagnoses in 2015 minus deaths in 2015 from pre-ART and ART | National Centre for HIV/AIDS, Dermatology and STIs (NCHADS) program data | VL <1,000 copies/mL Data from VL lab database. Calculated using a sample of people on ART receiving VL (65% PLHIV on ART tested for VL) | Medium |
| China^9^ | PEPFAR Regional Operational Plan | UNAIDS estimate | National Center for AIDS/STD Control and Prevention (NCAIDS) program data | NCAIDS program data | VL <1,000 copies/mL NCAIDS program data (viral load test for 90% of PLHIV on ART) | High |
| Colombia^10^ | UNAIDS report | UNAIDS estimate | Ministry of Health and Social protection data | UNAIDS Global AIDS response progress reporting | VL <1,000 copies/mL National program data | Medium |
| Cuba^11^ | WHO report | Ministry of Public Health, HIV Registry. Estimated as the difference between diagnosed and % of undiagnosed relative to the number of late diagnoses, ranging between 10% to 15% annually | HIV Registry (Calculated as everyone diagnosed between 1986 and 2012 minus deaths) | HIV Registry | Undetectable viral load HIV Registry | Medium |
| Denmark^6,12^ | Swedish-Danish HIV Cohort | UNAIDS estimate | National HIV surveillance reports 1995-2010 | Danish HIV Cohort Study (cohort includes all people living with HIV) | VL <500 copies/mL  Danish HIV Cohort Study | High |
| El Salvador^13^ | PEPFAR Regional Operational Plan | UNAIDS estimate | Source and quality unknown | Source and quality unknown | Source and quality unknown | Unknown |
| Estonia^14^ | National program review by WHO | Mid-value of UNAIDS estimate (7,200-11,000) | Based on newly registered HIV cases and AIDS deaths | Estonian Health Board - single central system for ARV drugs distribution | VL <200 copies/mL Estonian HIV-positive patients database | Medium |
| France^15^ | Cohort data from published study | Diagnosed + Undiagnosed (estimated using HIV incidence estimate and distribution of times from infection to diagnosis) | In care (estimated from French health insurance scheme) + not yet in care (estimated using size of the undiagnosed HIV population in 2010 and % of individuals aware of their HIV infection for >3 months among people living with HIV newly engaged in care from 2008-10) | Estimated using ART coverage rate among the FHDH-ANRS-CO4 cohort (Cohort includes data on 54,321 HIV-infected patients from 70 French general or university hospitals)  *FHDH - French Hospital Database on HIV | VL <50 copies/mL Estimated using viral suppression rate data from the FHDH-ANRS-CO4 cohort | Medium |
| Georgia^16^ | Published study | UNAIDS estimate | National AIDS health information system (AIDS HIS) | AIDS HIS | VL <1,000 copies/mL AIDS HIS | High |
| Guatemala^13^ | PEPFAR Regional Operational Plan | UNAIDS estimate | Source and quality unknown | Source and quality unknown | Source and quality unknown | Unknown |
| Guyana^17^ | UNAIDS country progress report | UNAIDS estimate | Numerator is unavailable | National AIDS Program Secretariat (NAPS) Care and Treatment data | VL <1,000 copies/mL National AIDS Program Secretariat (NAPS) Care and Treatment data | Low |
| Honduras^13^ | PEPFAR Regional Operational Plan | Source and quality unknown | Source and quality unknown | Source and quality unknown | Source and quality unknown | Unknown |
| Jamaica^18^ | Country report | UNAIDS estimate | HIV/AIDS Tracking System and the Treatment Site Databases | HIV/AIDS Tracking System and the Treatment Site Databases | Estimated using HIV/AIDS Tracking System and the Treatment Site Databases (5,885 viral load test) | Medium |
| Kazakhstan (15+)^19^ | PEPFAR Regional Operational Plan | UNAIDS estimate | Electronic HIV Case Management System (EHCMS) | EHCMS | VL <1,000 copies/mL  EHCMS (1,351 viral load test) | Medium |
| Kenya^20^ | PEPFAR Country Operational Plan | UNAIDS estimate | Numerator is unavailable | Site improvement monitoring system (SIMS) data | Estimated using data from SIMS | Low |
| Kyrgyzstan (15+)^19^ | PEPFAR Regional Operational Plan | UNAIDS estimate | Numerator is unavailable | Electronic HIV Case Management System (EHCMS) | VL <1,000 copies/mL  EHCMS (5,164 viral load test) | Low |
| Lao People’s Democratic Republic^21^ | UNAIDS country progress report | UNAIDS estimate | Data from Centre for HIV/AIDS and STI | Data from Centre for HIV/AIDS and STI | VL <1,000 copies/mL  Data from Centre for HIV/AIDS and STI | Medium |
| Malawi^22^ | PEPFAR Country Operational Plan | UNAIDS estimate | Numerator is unavailable | Source and quality unknown | Source and quality unknown | Low |
| Malaysia^23^ | WHO Country Fact Sheets | UNAIDS estimate | Numerator is unavailable | UNAIDS Global AIDS response progress reporting | VL <1,000 copies/mL  National program data | Low |
| Mauritius^24^ | UNAIDS country progress report | Estimate (Source and quality unknown) | National Day Care Centre for Immunosuppressed, Ministry of Health | ART Register, National Day Care Centre for Immunosuppressed, Ministry of Health | VL <1,000 copies/mL  Central Health Laboratory, Ministry of Health | Medium |
| Mexico^25^ | National program report | UNAIDS estimate | Centro Nacional para la prevención y el Control del VIH/SIDA (CENSIDA) - National register of HIV/AIDS cases | CENSIDA program data from public health sector institutions | Undetectable viral load  CENSIDA national program data | Medium |
| Mongolia^26^ | UNAIDS country progress report | UNAIDS estimate | Source and quality unknown | AIDS/STI Surveillance and Research Department of National Center for Communicable Diseases (NCCD) | VL <1,000 copies/mL  AIDS/STI Surveillance and Research Department of NCCD | Medium |
| Myanmar^27^ | WHO Country Fact Sheets | UNAIDS estimate | Numerator is unavailable | National program data (from Global AIDS response progress reporting) | National data. Calculated using a sample of people on ART receiving VL | Low |
| Namibia^28^ | PEPFAR Country Operational Plan | UNAIDS estimate | Numerator is unavailable | Source and quality unknown | Source and quality unknown | Low |
| Nepal^29^ | National program report | UNAIDS estimate | National program data | ART program data and HIV infection estimates | VL <1,000 copies/mL  National program data (National Public Health Laboratory) | Medium |
| Netherlands^30^ | Stichting HIV Monitoring | Mid-value of UNAIDS estimate (20,000-34,000) | Stichting HIV Monitoring (SHM) of people at Dutch HIV treatment centres | SHM data | VL <100 copies/mL SHM data | High |
| Nicaragua^31^ | UNAIDS country progress report | UNAIDS estimate | Ministry of Health records | ART therapy database | VL <1,000 copies/mL ART therapy database | Medium |
| Panama^13^ | PEPFAR Regional Operational Plan | UNAIDS estimate | Source and quality unknown | Source and quality unknown | Source and quality unknown | Unknown |
| Paraguay^32^ | UNAIDS country progress report | UNAIDS estimate | Dept. of comprehensive care/ National program of HIV/AIDS Control (PRONASIDA) | Dept. of comprehensive care/ National program of HIV/AIDS Control (PRONASIDA) | VL <1,000 copies/mL Dept. of comprehensive care/ PRONASIDA | Medium |
| Philippines^23^ | WHO Country Fact Sheets | UNAIDS estimate | HIV/AIDS & ART Registry of the Philippines, 2015 | HIV/AIDS & ART Registry of the Philippines, 2015 | VL <1,000 copies/mL  HIV/AIDS & ART Registry of the Philippines, 2015 | Medium |
| Romania^33^ | National program report | Source and quality unknown | Source and quality unknown | Source and quality unknown | Source and quality unknown | Unknown |
| Russia^34^ | Conference abstract | Estimate (Source and quality unknown) | Federal AIDS Centre database; national monitoring forms of Rospotrebnadzor (Russian Federal Service for Surveillance on Consumer Rights Protection and Human Wellbeing) | Federal AIDS Centre database; national monitoring forms of Rospotrebnadzor | VL <1,000 copies/mL Federal AIDS Centre database; national monitoring forms of Rospotrebnadzor | Medium |
| Rwanda^35^ | PEPFAR Country Operational Plan | UNAIDS estimate | Numerator is unavailable | Rwanda health management information system (HMIS) | Source and quality unknown | Low |
| South Africa^36^ | PEPFAR Country Operational Plan | National data from District Health Information System | Numerator is unavailable | National program data (from Global AIDS response progress reporting) | National Health Laboratory service electronic data | Low |
| Spain^37^ | National program report | UNAIDS estimate | EPI-ITS study (2005-2010) - a sentinel surveillance project in a network of clinics specializing in STI | Hospital survey | VL <500 copies/mL Hospital survey | Low |
| Sri Lanka^38^ | National program report | UNAIDS estimate | Estimate (Source and quality unknown) | National program data (from Global AIDS response progress reporting) | National data | Medium |
| Suriname (15+)^39,40^ | UNAIDS country progress report | UNAIDS estimate | National HIV master database, Monitoring & Evaluation unit, Ministry of Health | National HIV master database, Monitoring & Evaluation unit, Ministry of Health | VL <1,000 copies/mL National HIV master database, Monitoring & Evaluation unit, Ministry of Health | Medium |
| Swaziland^41^ | PEPFAR Country Operational Plan | Swaziland HIV Estimates and Projections 2015 | Source and quality unknown | Swaziland health management information system (SHIMS), Ministry of Health | SHIMS 2011; Viral Load Suppression of 85% | Medium |
| Sweden^42^ | Cohort data from published study | Diagnosed + undiagnosed (estimated based on HIV surveillance data reported to the Public Health Agency of Sweden) | Swedish InfCare HIV Cohort Study – includes 100 % of people diagnosed with HIV and linked to care | Swedish InfCare HIV Cohort Study | VL <200 copies/mL  Swedish InfCare HIV Cohort Study | High |
| Switzerland^43^ | Conference abstract | Diagnosed + undiagnosed (estimated based on MSM modeling study by Van Sighem et al. 2011 and assumptions about testing in other populations) | Back-calculated from people retained in care [Swiss HIV Cohort Study (SHCS) and non-SHCS patients in care] using 2010 data from European MSM Internet Survey | Swiss HIV Cohort Study (SHCS); survey among HIV care providers connected to the SHCS network; Swiss ART sales data to estimate the number of patients treated outside the SHCS network | VL <200 copies/mL Swiss HIV Cohort Study (SHCS); survey among HIV care providers connected to the SHCS network; estimate for patients treated outside the SHCS network | Low |
| Tajikistan (15+)^19^ | PEPFAR Regional Operational Plan | UNAIDS estimate | Electronic HIV Case Management System (EHCMS) | EHCMS | VL <1,000 copies/mL  EHCMS (357 people received viral load test) | Medium |
| Thailand^27^ | WHO Country Fact Sheets | UNAIDS estimate | National data | National Health Security Office (NHSO) and National AIDS Management Centre, Ministry of Public Health | VL <1,000 copies/mL NHSO and National AIDS Management Centre, Ministry of Public Health | Medium |
| Uganda^44^ | UNAIDS country progress report | UNAIDS estimate | Numerator is unavailable | District Health Information System (DHIS 2) | VL <1,000 copies/mL DHIS 2 | Low |
| Ukraine^45^ | National program data | UNAIDS estimate | Number of individuals informed as the HIV cases, excluding cumulatively registered number of AIDS deaths (routine government surveillance data) | Number of PLHIV on ART reported by AIDS Centers to Ukrainian Center for Disease Control (data from routine government surveillance data) | VL <40 copies/mL Estimation source is the number of blood samples tested for viral load | Medium |
| United Kingdom^46^ | National program report | Statistical model fitted to a range of surveillance and survey data | Statistical model fitted to a range of surveillance and survey data | National HIV surveillance data | VL <200 copies/mL National HIV surveillance data | Medium |
| United States^47^ | Centers for Disease Control and Prevention (CDC) surveillance report | National HIV Surveillance System. Estimated number of PLHIV aged ≥13 years | National HIV Surveillance System. Estimated number of PLHIV aged ≥13 years whose HIV infection had been diagnosed by year-end | Medical Monitoring Project. PLHIV aged ≥18 years who received medical care during Jan–April 2012 with documentation of ART prescription in the medical record | VL <200 copies/mL Medical Monitoring Project: Estimated number of PLHIV aged ≥18 years who received medical care during Jan–April 2011, and whose most recent VL in preceding 12 months was undetectable or <200 copies/mL | Medium |
| Zimbabwe^48^ | UNAIDS country progress report | UNAIDS estimate | Numerator is unavailable | Program data from electronic Patient Management System (ePMS) | VL <1,000 copies/mL Electronic Patient Management System (ePMS) data. Calculated using a sample of people on ART receiving VL (Targeted VL testing) | Low |

**Footnote:** Indicators are ranked according to the information below; continua are ranked according to the lowest graded indicator. If source of one indicator is unknown, the continuum is ranked according the grade of remaining three indicators. Quality of the continuum is ‘unknown’ if sources of the indicators PLHIV diagnosed, on ART and with viral suppression are unknown. The UNAIDS target “On ART” represents people taking ART which is usually defined by a surrogate marker such as filling pharmacy prescriptions.

*****National estimates and/or UNAIDS are high quality however it is important to remember that they are estimates and should be based on periodic surveillance data. Cascade methods should optimally reference the sources of data for the estimates.

**REFERENCES**

1. Dirección de Sida y ETS, Ministerio de Salud de la Nación. Boletin Sobre el VIH-SIDA en la Argentina. Numero 31 Ano XVII. Buenos Aires, Argentina. December 2014. Available from: <http://www.msal.gov.ar/images/stories/bes/graficos/0000000601cnt-2015-01-29_boletin-epidemiologico-vih-2014.pdf>.

2. Joint United Nations Programme on HIV/AIDS (UNAIDS). Regional Consultation on Re-targeting Universal Access to HIV Treatment in Eastern Europe and Central Asian Countries. Consultation Report. Istanbul, Turkey. April, 2014. Available from: [http://aids.md/aids/files/1435/EECA report on regional consultation on re-targeting_Eng.pdf](http://aids.md/aids/files/1435/EECA%20report%20on%20regional%20consultation%20on%20re-targeting_Eng.pdf).

3. The Kirby Institute. HIV, viral hepatitis and sexually transmissible infections in Australia Annual Surveillance Report 2014 HIV Supplement. The Kirby Institute, University of New South Wales, New South Wales, Australia. Available from: <http://kirby.unsw.edu.au/sites/default/files/hiv/resources/HIVASRsuppl2014_online.pdf>.

4. World Health Organization (WHO) Regional Office for Europe. HIV Programme Review in Belarus. Copenhagen, Denmark. November 2014. Available from: <http://www.euro.who.int/__data/assets/pdf_file/0010/273295/HIV-Programme-Review-in-Belarus.pdf?ua=1>.

5. Van Beckhoven D, Florence E, Ruelle J, et al. Good continuum of HIV care in Belgium despite weaknesses in retention and linkage to care among migrants. *BMC infectious diseases.* 2015;15(1):496.

6. Joint UN Programme on HIV/AIDS (UNAIDS). HIV estimates with uncertanity bounds 1990-2011. Available from: <http://www.unaids.org/en/resources/campaigns/20121120_globalreport2012/globalreport/>.

7. Ministry of Health of Brazil, Department of STI, AIDS and Viral Hepatitis. The Brazilian Experience: Treatment for All. Presented at AIDS 2016, Durban, South Africa.

8. U.S. President’s Emergency Plan for AIDS Relief (PEPFAR). Cambodia 2016 Country Operational Plan Strategic Direction Summary. Washington DC, United States. Available from: <http://www.pepfar.gov/documents/organization/257617.pdf>.

9. U.S. President’s Emergency Plan for AIDS Relief (PEPFAR). Asia Regional Program 2016 Regional Operational Plan Strategic Direction Summary. Washington DC, United States. Available from: <http://www.pepfar.gov/documents/organization/257358.pdf>.

10. Joint UN Programme on HIV/AIDS (UNAIDS). 90-90-90 An ambitious treatment target to help end the AIDS epidemic. Geneva, Switzerland 2014. Available from: <http://www.unaids.org/sites/default/files/media_asset/90-90-90_en_0.pdf>.

11. Pan American Health Organization (PAHO). Antiretroviral Treatment in the Spotlight: A Public Health Analysis in Latin America and the Caribbean 2013. Washington, DC, United States. Available from: <http://www.paho.org/Hq/index.php?option=com_docman&task=doc_view&gid=23710&Itemid=>.

12. Helleberg M, Haggblom A, Sonnerborg A, Obel N. HIV care in the Swedish-Danish HIV cohort 1995-2010, closing the gaps. *PloS one.* 2013;8(8):e72257.

13. U.S. President’s Emergency Plan for AIDS Relief (PEPFAR). Central America Region 2016 Regional Operational Plan Strategic Direction Summary. Washington DC, United States. Available from: <http://www.pepfar.gov/documents/organization/257655.pdf>.

14. World Health Organization (WHO) Regional Office for Europe. HIV/AIDS treatment and care in Estonia - Evaluation Report. Copenhagen, Denmark. June, 2014. Available from: <http://www.euro.who.int/__data/assets/pdf_file/0008/255671/HIVAIDS-treatment-and-care-in-Estonia.pdf>.

15. Supervie V, Marty L, Lacombe JM, Dray-Spira R, Costagliola D, group F-ACs. Looking beyond the cascade of HIV care to end the AIDS epidemic: estimation of the time interval from HIV infection to viral suppression. *Journal of acquired immune deficiency syndromes.* Jun 27 2016.

16. Chkhartishvili N, Chokoshvili O, Dvali N, Abutidze A, Sharvadze L, Tsertsvadze T. Significant Improvements Are Needed in HIV Care Continuum to Meet 90-90-90 Targets in Georgia. *Journal of the International Association of Providers of AIDS Care.* Sep 14 2016.

17. Presidential Commission on HIV and AIDS, Guyana. UNAIDS AIDS Response Progress Report 2014. March, 2015. Available from: <http://www.unaids.org/sites/default/files/country/documents/GUY_narrative_report_2015.pdf>.

18. National HIV/STI Programme, Ministry of Health, Jamaica. HIV Epidemiological Profile, 2014. Available from: <http://moh.gov.jm/wp-content/uploads/2015/07/2014-HIV-Epi-Update.pdf>.

19. U.S. President’s Emergency Plan for AIDS Relief (PEPFAR). Central Asia Region 2016 Regional Operational Plan Strategic Direction Summary. Washington DC, United States. Available from: <http://www.pepfar.gov/documents/organization/257618.pdf>.

20. U.S. President’s Emergency Plan for AIDS Relief (PEPFAR). Kenya 2016 Country Operational Plan Strategic Direction Summary. Washington DC, United States. Available from: <http://www.pepfar.gov/documents/organization/257644.pdf>.

21. Joint United Nations Programme on HIV/AIDS (UNAIDS). Lao PDR Global AIDS Response Progress Country Report, 2015. Vientiane, Lao PDR. March, 2015. Available from: <http://www.unaids.org/sites/default/files/country/documents/LAO_narrative_report_2015.pdf>.

22. U.S. President’s Emergency Plan for AIDS Relief (PEPFAR). Malawi 2016 Country Operational Plan Strategic Direction Summary. Washington DC, United States. Available from: <http://www.pepfar.gov/documents/organization/257638.pdf>.

23. WHO Western Pacific Region. Cascade of HIV testing, care and treatment services, 2013 & 2014: selected country profiles. Available from: <http://iris.wpro.who.int/bitstream/handle/10665.1/12925/WPR_2016_DCD_001_eng.pdf>.

24. Joint United Nations Programme on HIV/AIDS (UNAIDS). Country Progress Report 2015 Republic of Mauritius. April, 2015. Available from: <http://www.unaids.org/sites/default/files/country/documents/MUS_narrative_report_2015.pdf>.

25. Centro Nacional para la prevención y el Control del VIH/SIDA (CENSIDA). Cascada del Continuo de la Atencion del VIH: Total, Secretaria de Salud y Poblaciones Claves. Mexico, 2015. Available from: <http://www.paho.org/hq/index.php?option=com_docman&task=doc_view&Itemid=270&gid=34367&lang=pt>.

26. Joint United Nations Programme on HIV/AIDS (UNAIDS). AIDS Response Progress Report. Mongolia 2014. March, 2015. Available from: <http://www.unaids.org/sites/default/files/country/documents/MNG_narrative_report_2015.pdf>.

27. WHO South East Asia Region. HIV/AIDS Fact Sheets from 10 Member States. December, 2015. Available from: <http://www.searo.who.int/entity/hiv/data/factsheets/en/>.

28. U.S. President’s Emergency Plan for AIDS Relief (PEPFAR). Namibia 2016 Country Operational Plan Strategic Direction Summary. Washington DC, United States. Available from: <http://www.pepfar.gov/documents/organization/257636.pdf>.

29. National Centre for AIDS and STD Control, Government of Nepal. Factsheets of HIV Program in Nepal. July 2016. Available from: [http://www.ncasc.gov.np/uploaded/facts_n_figure/NCASC_Infographics_2016_Final_Optimized for Web.pdf](http://www.ncasc.gov.np/uploaded/facts_n_figure/NCASC_Infographics_2016_Final_Optimized%20for%20Web.pdf).

30. Ard van Sighem LG, Colette Smit, Ineke Stolte, Peter Reiss. Monitoring Programme report 2014: Human Immunodeficiency Virus (HIV) infection in the Netherland. Stichting HIV Monitoring (SHM). Amsterdam, The Netherlands. November, 2014. Available from: <http://www.hiv-monitoring.nl/english/>.

31. Gobierno de Reconciliacion y Unidad Nacional. Informe global sobre la epidemia del SIDA Nicaragua 2015. UNAIDS Country progress report, March 2015. Available from: <http://www.unaids.org/sites/default/files/country/documents/NIC_narrative_report_2015.pdf>.

32. Ministerio de Salud Pública y Bienestar Social, Programa Nacional de Control del VIH/sida/ITS, Paraguay. Informe de la Situación Epidemiológica del VIH y Sida 2014. March, 2015. UNAIDS Country progress report 2015. Available from: <http://www.unaids.org/sites/default/files/country/documents/PRY_narrative_report_2015.pdf>.

33. Comisia Nationala de Lupta Anti-SIDA (CNLAS). HIV/AIDS in Romania. December 2015 Update. Available from: <http://www.cnlas.ro/images/doc/01122015_rom.pdf>

34. Pokrovskaya A, Popova A, Ladnaya N, Yurin O. The cascade of HIV care in Russia, 2011-2013. *Journal of the International AIDS Society.* 2014;17(4 Suppl 3):19506.

35. U.S. President’s Emergency Plan for AIDS Relief (PEPFAR). Rwanda 2016 Country Operational Plan Strategic Direction Summary. Washington DC, United States. Available from: <http://www.pepfar.gov/documents/organization/257633.pdf>.

36. U.S. President’s Emergency Plan for AIDS Relief (PEPFAR). South Africa 2016 Country Operational Plan Strategic Direction Summary. Washington DC, United States. Available from: <http://www.pepfar.gov/documents/organization/257632.pdf>.

37. Grupo de Trabajo sobre Tratamientos del VIH. GeSIDA 2014, Espana: Cascada del tratamiento del VIH en España. Málaga, Spain. November, 2014. Available from: <http://gtt-vih.org/actualizate/la_noticia_del_dia/27-11-14>.

38. Presidential address 2015: Sexual health in the era of HIV. Sri Lanka Journal of Sexual Health and HIV Medicine, 2015.

39. Ministry of Health, Suriname. UNAIDS AIDS Response Progress Report 2015. March, 2015. Available from: <http://www.unaids.org/sites/default/files/country/documents/SUR_narrative_report_2015.pdf>.

40. UNAIDS. AIDSinfo Online Database. Geneva, Switzerland, 2015. Available from: <http://www.aidsinfoonline.org/devinfo/libraries/aspx/Home.aspx>.

41. U.S. President’s Emergency Plan for AIDS Relief (PEPFAR). Swaziland 2016 Country Operational Plan Strategic Direction Summary. Washington DC, United States. Available from: <http://www.pepfar.gov/documents/organization/257630.pdf>.

42. Gisslen M, Svedhem V, Lindborg L, et al. Sweden, the first country to achieve the Joint United Nations Programme on HIV/AIDS (UNAIDS)/World Health Organization (WHO) 90-90-90 continuum of HIV care targets. *HIV medicine.* Aug 18 2016.

43. Philipp Kohler AJS, Bruno Ledergerber, Pietro L. Vernazza. Estimates of HIV Prevalence, Proportion of Diagnosed Patients and Quality of Treatment in Switzerland. Conference on Retroviruses and Opportunistic Infections. February, 2015. Seattle, WA, United States. Abstract 1008, 2015.

44. The Republic of Uganda. The HIV and AIDS Uganda UNAIDS Country Progress Report 2014. June, 2015. Available from: <http://www.unaids.org/sites/default/files/country/documents/UGA_narrative_report_2015.pdf>.

45. Y. Sereda, M. Nikolko, I. Shvab. HIV treatment cascade in Ukraine: a cross-sectional analysis of regional patterns in 2015. Presented at AIDS 2016, Durban, South Africa.

46. Skingsley A, Yin Z, Kirwan P, Croxford S, Chau C, Conti S, Presanis A, Nardone A, Were J, Ogaz D, Furegato M, Hibbert M, Aghaizu A, Murphy G, Tosswill J, Hughes G, Anderson J, Gill ON, Delpech VC and contributors. HIV in the UK – Situation Report 2015: data to end 2014. November 2015. Public Health England, London. Available from: https://<http://www.gov.uk/government/uploads/system/uploads/attachment_data/file/477702/HIV_in_the_UK_2015_report.pdf>

47. Centers for Disease Control and Prevention. Monitoring selected national HIV prevention and care objectives by using HIV surveillance data—United States and 6 dependent areas—2013. HIV Surveillance Supplemental Report 2015; 20 (No. 2). Atlanta, GA, United States. July 2015. Available from: <http://www.cdc.gov/hiv/pdf/library/reports/surveillance/cdc-hiv-surveillancereport_vol20_no2.pdf>.

48. Joint United Nations Programme on HIV/AIDS (UNAIDS). Global AIDS Response Progress Report. Zimbabwe 2015. Available from: <http://www.unaids.org/sites/default/files/country/documents/ZWE_narrative_report_2015.pdf>.
