# Supplementary material for: Status and methodology of publicly available national HIV care continua and 90-90-90 targets: A systematic review
Source: PLoS Med. 2017 Apr 4;14(4):e1002253. doi: 10.1371/journal.pmed.1002253 (PMC5380306; doi:10.1371/journal.pmed.1002253)
Supplement: S2 Table — (DOCX) [file pmed.1002253.s003.docx]

**Supporting Information**

**Table 2**: Summary and grading of the sources of information on the four key steps in the HIV continua of care for 29 countries, which do not have estimates on viral suppression.

**KEY**

| **COLOUR** | **Estimated PLHIV** | **PLHIV Diagnosed** | **PLHIV on ART** | **PLHIV with Viral Suppression** |
| --- | --- | --- | --- | --- |
| High quality | - National estimates* - UNAIDS estimate (Spectrum)* - Survey or surveillance data | – National program data  – National cohort data | – National program data  – National cohort data | Individual viral load data for everyone on ART from program database; surveys of patient cohort representative of everyone on ART; nationally-representative population surveys |
| Medium quality | Estimates based on sub-national/sub-population data | Estimates based on surveillance estimates and/or diagnosis in select sub-population | ART coverage estimated using sub-population cohort or surveillance | Estimates based on sub-sample of those on ART or surveillance/ surveys |
| Low quality | - Estimates based on modeling studies  - Numerator is unavailable | Estimates derived from non-representative selection of clinics and/or hospitals | Coverage based on non-representative selection of clinics and/or hospitals | Estimates from non-representative selection of clinics and/or hospitals |
|  | Source and quality unknown | | | |

PLHIV – people living with HIV

| **COUNTRY** | **SOURCE** | **ESTIMATED PEOPLE LIVING WITH HIV** | **PEOPLE LIVNG WITH DIAGNOSED HIV** | **PEOPLE RECEIVING ART** | **PEOPLE ON ART WITH SUPPRESSED VIRAL LOAD** |
| --- | --- | --- | --- | --- | --- |
| Angola^1^ | PEPFAR Country Operational Plan | UNAIDS estimate | Numerator is unavailable | National AIDS Institute (INLS) program data | Numerator is unavailable |
| Antigua and Barbuda^2^ | Newspaper article | Numerator is unavailable | National program data | National program data | Numerator is unavailable |
| Bahamas^3^ | PEPFAR Regional Operational Plan | UNAIDS estimate | Numerator is unavailable | Source and quality unknown | Numerator is unavailable |
| Bangladesh^4^ | WHO Country Fact Sheets | UNAIDS estimate | Estimated using cumulative number of people diagnosed with HIV | National programme data (from Global AIDS response progress reporting) | Numerator is unavailable |
| Barbados^3^ | PEPFAR Regional Operational Plan | Surveillance data | Numerator is unavailable | Source and quality unknown | Numerator is unavailable |
| Bhutan^5^ | UNAIDS country progress report | UNAIDS estimate | National program PLHIV database | National program PLHIV database | Numerator is unavailable |
| Botswana^6^ | PEPFAR Country Operational Plan | UNAIDS estimate | Numerator is unavailable | Source and quality unknown | Numerator is unavailable |
| Burundi^7^ | PEPFAR Country Operational Plan | UNAIDS estimate | Numerator is unavailable | National AIDS Council (CNLS) program data | Numerator is unavailable |
| Cameroon^8^ | PEPFAR Country Operational Plan | UNAIDS estimate | Numerator is unavailable | Source and quality unknown | Numerator is unavailable |
| Cote d’Ivoire^9^ | PEPFAR Country Operational Plan | UNAIDS estimate | Numerator is unavailable | PEPFAR Cote d'Ivoire program data | Numerator is unavailable |
| Dominican Republic^10^ | PEPFAR Country Operational Plan | UNAIDS estimate | Numerator is unavailable | HIV patient monitoring system | Numerator is unavailable |
| Egypt^11^ | UNAIDS country progress report | UNAIDS estimate | HIV case detection | Source and quality unknown | Numerator is unavailable |
| Ethiopia^12^ | PEPFAR Country Operational Plan | HIV-related estimates and projections for Ethiopia–Federal Ministry of Health | Numerator is unavailable | Source and quality unknown | Numerator is unavailable |
| Ghana^13^ | PEPFAR Country Operational Plan | UNAIDS estimate | Numerator is unavailable | National AIDS Control Programme (NACP) Service Data | Numerator is unavailable |
| Haiti^14^ | PEPFAR Country Operational Plan | Small Area Estimates, Office of Global AIDS Coordinator 2015 | Numerator is unavailable | National program data | Numerator is unavailable |
| India^4^ | WHO Country Fact Sheets | UNAIDS estimate | Estimated using cumulative number of people diagnosed with HIV | National programme data (from Global AIDS response progress reporting) | Numerator is unavailable |
| Indonesia^15^ | PEPFAR Country Operational Plan | UNAIDS estimate | Numerator is unavailable | Ministry of Health data | Numerator is unavailable |
| Lesotho^16^ | PEPFAR Country Operational Plan | UNAIDS estimate | Numerator is unavailable | National program data (from Global AIDS response progress reporting) | Numerator is unavailable |
| Morocco^17^ | UNAIDS country progress report | Data not available | Source and quality unknown | Source and quality unknown | Numerator is unavailable |
| Mozambique^18^ | PEPFAR Country Operational Plan | UNAIDS estimate | Numerator is unavailable | National program data | Numerator is unavailable |
| Nigeria^19^ | PEPFAR Country Operational Plan | Estimate based on PEPFAR Datapack | Numerator is unavailable | National program data (from Global AIDS response progress reporting) | Numerator is unavailable |
| Papua New Guinea^20^ | PEPFAR Country Operational Plan | UNAIDS estimate | Numerator is unavailable | National program data (from Global AIDS response progress reporting) | Numerator is unavailable |
| Somalia^21^ | UNAIDS country progress report | UNAIDS estimate | Program data from 3 Somali zones | Program data | Numerator is unavailable |
| South Sudan^22^ | PEPFAR Country Operational Plan | UNAIDS estimate | Numerator is unavailable | National program data | Numerator is unavailable |
| Tanzania^23^ | PEPFAR Country Operational Plan | UNAIDS estimate | Numerator is unavailable | National program data | Numerator is unavailable |
| Timor-Leste^24^ | UNAIDS country progress report | UNAIDS estimate | Numerator is unavailable | National program data | Numerator is unavailable |
| Trinidad^3^ | PEPFAR Regional Operational Plan | Estimate (Source and quality unknown) | Numerator is unavailable | Source and quality unknown | Numerator is unavailable |
| Viet Nam^25^ | PEPFAR Country Operational Plan | UNAIDS estimate | Viet Nam Administration of HIV/AIDS Control (VAAC) HIV case reporting data | Viet Nam Administration of HIV/AIDS Control (VAAC) program data | Numerator is unavailable |
| Zambia^26^ | PEPFAR Country Operational Plan | Estimate based on PEPFAR Datapack | Numerator is unavailable | Source and quality unknown | Numerator is unavailable |

**Footnote:** Indicators are ranked according to the information below. The UNAIDS target “On ART” represents people taking ART which is usually defined by a surrogate marker such as filling pharmacy prescriptions.

*****National estimates and/or UNAIDS are high quality however it is important to remember that they are estimates and should be based on periodic surveillance data. Cascade methods should optimally reference the sources of data for the estimates.

**REFERENCES**

1. U.S. President’s Emergency Plan for AIDS Relief (PEPFAR). Angola 2016 Country Operational Plan Strategic Direction Summary. Washington DC, United States. Available from: <http://www.pepfar.gov/documents/organization/257356.pdf>.

2. Caribtimes. Antigua & Barbuda HIV Treatment Cascade: Filling the Gaps to Reduce the Number of People Contracting HIV and the Number of People Dying From AIDS. December, 2014. Available from: [http://caribtimes.com/2014/12/01/antigua-barbuda-hiv-treatment-cascade-filling-the-gaps-to-reduce-the-number-of-people-contracting-hiv-and-the-number-of-people-dying-from-aids/ - .VbFQCCqqqko](http://caribtimes.com/2014/12/01/antigua-barbuda-hiv-treatment-cascade-filling-the-gaps-to-reduce-the-number-of-people-contracting-hiv-and-the-number-of-people-dying-from-aids/#.VbFQCCqqqko).

3. U.S. President’s Emergency Plan for AIDS Relief (PEPFAR). Caribbean Region 2016 Regional Operational Plan Strategic Direction Summary. Washington DC, United States. Available from: <http://www.pepfar.gov/documents/organization/257656.pdf>.

4. WHO South East Asia Region. HIV/AIDS Fact Sheets from 10 Member States. December, 2015. Available from: <http://www.searo.who.int/entity/hiv/data/factsheets/en/>.

5. National AIDS Control Progranmme, Department of Public Health Ministry of Health. UNAIDS Country Progress Report on the HIV Response in Bhutan-2015. Thimpu, Bhutan. April, 2015. Available from: <http://www.unaids.org/sites/default/files/country/documents/BTN_narrative_report_2015.pdf>.

6. U.S. President’s Emergency Plan for AIDS Relief (PEPFAR). Botswana 2016 Country Operational Plan Strategic Direction Summary. Washington DC, United States. Available from: <http://www.pepfar.gov/documents/organization/257359.pdf>.

7. U.S. President’s Emergency Plan for AIDS Relief (PEPFAR). Burundi 2016 Country Operational Plan Strategic Direction Summary. Washington DC, United States. Available from: <http://www.pepfar.gov/documents/organization/257659.pdf>.

8. U.S. President’s Emergency Plan for AIDS Relief (PEPFAR). Cameroon 2016 Country Operational Plan Strategic Direction Summary. Washington DC, United States. Available from: <http://www.pepfar.gov/documents/organization/257657.pdf>.

9. U.S. President’s Emergency Plan for AIDS Relief (PEPFAR). Côte d'Ivoire 2016 Country Operational Plan Strategic Direction Summary. Washington DC, United States. Available from: <http://www.pepfar.gov/documents/organization/257653.pdf>.

10. U.S. President’s Emergency Plan for AIDS Relief (PEPFAR). Dominican Republic 2016 Country Operational Plan Strategic Direction Summary. Washington DC, United States. Available from: <http://www.pepfar.gov/documents/organization/257651.pdf>.

11. Joint United Nations Programme on HIV/AIDS (UNAIDS). National HIV Programme Situation and Gap Analysis in Egypt. April, 2015. Available from: <http://www.unaids.org/sites/default/files/country/documents/EGY_narrative_report_2015.pdf>.

12. U.S. President’s Emergency Plan for AIDS Relief (PEPFAR). Ethiopia 2016 Country Operational Plan Strategic Direction Summary. Washington DC, United States. Available from: <http://www.pepfar.gov/documents/organization/257650.pdf>.

13. U.S. President’s Emergency Plan for AIDS Relief (PEPFAR). Ghana 2016 Country Operational Plan Strategic Direction Summary. Washington DC, United States. Available from: <http://www.pepfar.gov/documents/organization/257649.pdf>.

14. U.S. President’s Emergency Plan for AIDS Relief (PEPFAR). Haiti 2016 Country Operational Plan Strategic Direction Summary. Washington DC, United States. Available from: <http://www.pepfar.gov/documents/organization/257647.pdf>.

15. U.S. President’s Emergency Plan for AIDS Relief (PEPFAR). Indonesia 2016 Country Operational Plan Strategic Direction Summary. Washington DC, United States. Available from: <http://www.pepfar.gov/documents/organization/257645.pdf>.

16. U.S. President’s Emergency Plan for AIDS Relief (PEPFAR). Lesotho 2016 Country Operational Plan Strategic Direction Summary. Washington DC, United States. Available from: <http://www.pepfar.gov/documents/organization/257640.pdf>.

17. Kingdom of Morocco. Mise en œuvre de la déclaration politique sur le VIH/SIDA.UNAIDS Country progress report 2015. March, 2015. Available from: <http://www.unaids.org/sites/default/files/country/documents/MAR_narrative_report_2015.pdf>.

18. U.S. President’s Emergency Plan for AIDS Relief (PEPFAR). Mozambique 2016 Country Operational Plan Strategic Direction Summary. Washington DC, United States. Available from: https://<http://www.pepfar.gov/documents/organization/257637.pdf>.

19. U.S. President’s Emergency Plan for AIDS Relief (PEPFAR). Nigeria 2016 Country Operational Plan Strategic Direction Summary. Washington DC, United States. Available from: <http://www.pepfar.gov/documents/organization/257635.pdf>.

20. U.S. President’s Emergency Plan for AIDS Relief (PEPFAR). Papua New Guinea 2016 Country Operational Plan Strategic Direction Summary. Washington DC, United States. Available from: <http://www.pepfar.gov/documents/organization/257634.pdf>.

21. Joint United Nations Programme on HIV/AIDS (UNAIDS). Progress report for Somalia HIV and AIDS Response 2014. June 2015. Available from: <http://www.unaids.org/sites/default/files/country/documents/SOM_narrative_report_2015.pdf>.

22. U.S. President’s Emergency Plan for AIDS Relief (PEPFAR). South Sudan 2016 Country Operational Plan Strategic Direction Summary. Washington DC, United States. Available from: <http://www.pepfar.gov/documents/organization/257631.pdf>.

23. U.S. President’s Emergency Plan for AIDS Relief (PEPFAR). Tanzania 2016 Country Operational Plan Strategic Direction Summary. Washington DC, United States. Available from: https://<http://www.pepfar.gov/documents/organization/257629.pdf>.

24. National AIDS Programme, Ministry of Health, Timor-Leste. UNAIDS Global AIDS Response Progress Report 2015. Dili, Timor-Leste. June, 2015. Available from: <http://www.unaids.org/sites/default/files/country/documents/TLS_narrative_report_2015.pdf>.

25. U.S. President’s Emergency Plan for AIDS Relief (PEPFAR). Viet Nam 2016 Country Operational Plan Strategic Direction Summary. Washington DC, United States. Available from: <http://www.pepfar.gov/documents/organization/257625.pdf>.

26. U.S. President’s Emergency Plan for AIDS Relief (PEPFAR). Zambia 2016 Country Operational Plan Strategic Direction Summary. Washington DC, United States. Available from: <http://www.pepfar.gov/documents/organization/257624.pdf>.
